# Effects of rehydration and food consumption on salivary flow, pH and buffering capacity in young adult volunteers during ergometer exercise

**DOI:** 10.1186/1550-2783-10-49

**Published:** 2013-10-28

**Authors:** Mai Tanabe, Toshiyuki Takahashi, Kazuhiro Shimoyama, Yukako Toyoshima, Toshiaki Ueno

**Affiliations:** 1 Department of Sports Medicine/Dentistry, Graduate School of Medical and Dental Sciences, Tokyo Medical and Dental University, Bunkyo-ku, Japan; 2 Oral health care science, Tokyo Medical and Dental University, Bunkyo-ku, Japan; 3 Japanese Institute of Sports Science, Kita-ku, Japan

**Keywords:** Saliva, Rehydration, Sports drink, Exercise, Oral care

## Abstract

**Background:**

The aim of this study was to investigate the influences of rehydration and food consumption on salivary flow, pH, and buffering capacity during bicycle ergometer exercise in participants.

**Methods:**

Ten healthy volunteers exercised on a bicycle ergometer at 80% of their maximal heart rate. These sessions lasted for two periods of 20 min separated by 5-min rest intervals. Volunteers were subjected to one of the following conditions: (1) no water (mineral water) or food consumption, (2) only water for rehydration, (3) water and food consumption, (4) a sports drink only for rehydration, and (5) rehydration with a sports drink and food. Statistical significance was assessed using one-way analysis of variance and Dunnett’s test (*p* < 0.05).

**Results:**

The salivary pH decreased significantly during and after exercise in conditions 4 and 5. The salivary buffering capacity decreased significantly during exercise and/or after the exercise in conditions 1, 3, 4, and 5.

**Conclusions:**

The results showed that salivary pH and buffering capacity decreased greatly depending on the combination of a sports drink and food.

## Background

It was reported that the decayed, missing, and filled teeth index and the risk of tooth erosion in athletes is relatively high as compared with that of ordinary people [1-3]. The mouth should be functional and free from disease, facilitating good nutrition and physical wellbeing to achieve maximum sporting potential [2]. In general, dental caries and erosion seen in athletes are thought to be caused by dry mouth with exercise-induced rehydration, large consumption of sports drinks and foods, and poor brushing of teeth because of physical fatigue. Among them, the sensation of dry mouth and dehydration means a decrease in the salivary flow rate, which causes a decline in the irrigation function in the oral environment. Many studies have also shown that a decrease in salivary secretion causes a decline in oral sugar clearance capacity in patients with dry mouth symptoms. A previous study in our laboratory reported that treadmill and ergometer exercises induced decreases of both the salivary flow rate and the salivary buffering capacity [4-6]. Thus, a decrease of salivary secretion indicates an increase in the risk of dental caries and erosion [4,7,8]. In addition, in many studies regarding the risk of dental caries and erosion, salivary secretion, salivary pH, and salivary buffering capacity were used as the parameters. Hirose et al. indicated that significant positive correlations were noted between salivary flow rate and salivary pH, but positive correlations were not noted between salivary flow rate and salivary buffering capacity [9]. If the pH of saliva is <5.5, the critical pH of dental enamel, then the mineral of dental enamel tends to dissolve [10]. Therefore, using the salivary pH and salivary buffering capacity to discuss dental caries and erosion is important.

However, many athletes were observed drinking isotonic and/or soft drinks that contained high acid and/or sugar contents, which resulted in an increased risk of dental caries and erosion. Drinking water during exercise can prevent excessive dehydration and changes in electrolyte balance, and can maintain the salivary secretion function [11]. Peter et al. studied the effects of rehydration on performance following moderate dehydration, and found that constituents other than water, simple transportable monosaccharides and sodium, are important for maximal exercise performance and effective recovery associated with endurance exercise-induced dehydration [12].

Moreover, people commonly consume foods such as fruits and supplements during exercise. Studies have reported that salivary pH values immediately increase after food consumption [13]. However, the influence on the oral environment of exercise with water and nutritional support is unclear. In the present study, we investigated the influences of rehydration and food consumption on salivary flow, pH, and buffering capacity during bicycle ergometer exercise in healthy volunteer participants.

## Methods

Experiments were performed on 10 healthy volunteers [4 females, mean ± standard deviation (SD) age, height, and weight: 20.5 ± 1.1 years, 160.5 ± 3.8 cm, and 55.7 ± 4.3 kg, respectively; 6 males, mean ± SD of age, height, and weight: 23.0 ± 3.1 years, 175.6 ± 7.47 cm, and 65.3 ± 4.3 kg, respectively]. The volunteers were fully dentate and had no oral disorders or braces. This study was approved by the Ethical Committee for Human Research of the Faculty of Dentistry, Tokyo Medical and Dental University, and the participants provided informed consent according to institutional guidelines.

The test was started at least 2 h after the last meal and at least 1 h after brushing the teeth [4-6]. The test exercise on the bicycle ergometer (Aerobike Ai, Combi Wellness Corporation, Tokyo, Japan) consisted of a warm-up of 5–10 min, a 20-min aerobic exercise at the test intensity determined to be 80% of the maximal heart rate, a warm-down exercise (1 min), 10-min rest, and repetition of the first warm-up/exercise cycle. The ergometer recorded the heart rate in real time from a sensor attached to the earlobe. The load of the pedal for exercise was automatically controlled by the ergometer at an intensity from level 1 to level 20, determined by the heart rate, and the pedal did not allow freewheeling.

Each volunteer tested the five conditions on different days in a random order. The fluid intake was at each participant’s discretion during exercise, but the food intake was assigned in the resting period (jelly-type nutritional supplement) and just after the exercise (banana). The conditions were as follows: (1) no intake of fluid or food, (2) intake of mineral water, (3) intake of mineral water and food (jelly-type nutritional supplement and banana), (4) intake of sports drink, and (5) intake of sports drink and food.

We used mineral water (Evian, Danone Waters of Japan Co., Tokyo, Japan) and a sports drink (Aquarius, Coca-Cola & Co., Ltd., Tokyo, Japan) as the sources of the fluid intake. Aquarius is one of the major sports drink brands in Japan. We used a jelly-type nutritional supplement (Wider In Jerry, Morinaga & Co., Ltd., Tokyo, Japan) and bananas (mean weight 147.9 ± 18.0 g) as the sources of food.

Salivary production was stimulated by chewing a piece of unflavored paraffin wax for 3 min and 30 s. After 30 s of prestimulation, whole saliva samples were collected in a container for 3 min. The volume of the stimulated whole saliva samples was measured. Whole saliva samples were collected before, during, and after exercise. Salivary pH and buffering capacity were measured using a hand-held pH meter (Checkbuf^TM^, Horiba Ltd., Tokyo, Japan) [4-6]. Calibration of the pH meter was done for each participant and each test with usage of dedicated standard pH-4.0 and pH-7.0 solutions. Salivary pH was directly measured from 0.25 ml of a saliva sample placed on the electrode sensor of the pH meter. To examine the salivary buffering capacity, 0.25 ml of dedicated lactic acid solution (pH 3.0) was dripped into the saliva sample on the electrode sensor. The pH meter was gently shaken for 20 s to mix the saliva sample and the lactic acid solution.

The statistical significance of the results was assessed using one-way analysis of variance and Dunnett’s test. For all the statistical analyses, *p*-values of <0.05 were considered significant.

## Results

In conditions 2 and 4, the mean quantities of the mineral water consumed were 642.4 ± 230.1 ml and 630.1 ± 188.7 ml, respectively. In conditions 3 and 5, those of the sports drink were 751.0 ± 152.9 ml and 714.0 ± 155.6 ml, respectively. No significant difference was present between the two groups.

**Figure 1:**
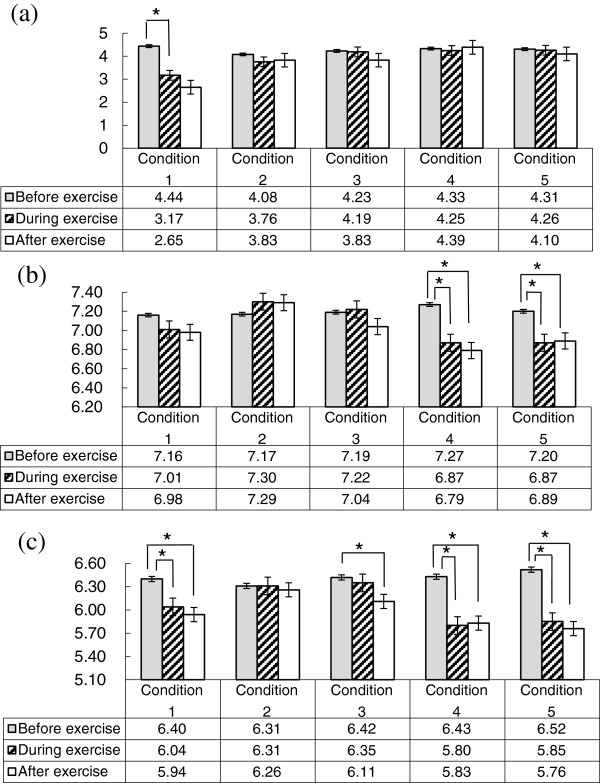
**Changes of salivary flow rate (a), salivary pH (b) and salivary buffering capacity (c).** Numerical values in table are the means of 10 participants.

Figure 1(a) shows the salivary flow rates. In condition 1, the salivary flow rate after exercise decreased by 40.3% compared with that before exercise (*p* < 0.05). In the other conditions, there was no significant difference in the salivary flow rate or its variations during the experiment.

Figure 1(b) shows the changes of salivary pH. In condition 4, salivary pH during and after exercise significantly decreased by 5.5% and 6.6%, respectively, compared with before exercise, and in condition 5, salivary pH during and after exercise significantly decreased by 4.6% and 4.3%, respectively, compared with before exercise. In condition 2, salivary pH during and after exercise did not decrease compared with that before exercise.

Figure 1(c) shows the changes of salivary buffering capacity. In condition 1, salivary buffering capacity during and after exercise significantly decreased by 5.6% and 7.2%, respectively, compared with before exercise. In condition 4, salivary buffering capacity during and after exercise significantly decreased by 9.8% and 9.3%, respectively, compared with before exercise. In condition 5, salivary buffering capacity during and after exercise significantly decreased by 10.3% and 11.7%, respectively, compared with before exercise. In condition 3, salivary buffering capacity after exercise significantly decreased by 4.8% compared with before exercise. In condition 2, salivary buffering capacity was almost constant throughout the experiment.

## Discussion

The mean stimulated salivary flow rate induced by chewing was reported to be 1.6 ml/min [7]. In the present study, the mean salivary flow rate after exercise was 0.77 ml/min in condition 1. Salivary secretion is strongly affected by the neural control of the autonomic nervous system, which indirectly regulates the salivary flow rate. The salivary flow rate depends on the autonomic state [14]. Because an increase of sympathetic activation is caused by sports and exercise, active exercise was expected to decrease the salivary flow rate [15]. Comparing the salivary secretion function of mineral water and the sports drink, the sports drink had a stronger inhibitory action on salivary secretion than mineral water. The taste of the sports drink is thought to bring about a difference in the quantity of the fluid intake during sports and exercise [4]. The results of the present study indicate that adequate hydration during sports and exercise inhibited the decrease of the salivary secretion function and the risk of dental caries and erosion.

Carbonic acid is known to decrease blood pH, and salivary secretion always requires adequate supply of nutrients from the blood [16,17]. The change in salivary pH depends on the level of CO_2_ in the blood [17]. With an increase in the blood CO_2_ level, CO_2_ is transferred from the blood to the saliva at a higher rate, with a subsequent decrease in salivary pH [14]. This function could explain the decrease in salivary pH during and after exercise compared with before exercise in condition 1. Nakano et al. studied the effects of exercise on salivary flow rate and buffering capacity, and found that exercise was a significant factor decreasing both salivary flow rate and the buffering capacity, in line with our results [5,6].

Many sports drinks contain acids such as citric acid, which increases the voluntary consumption of sports drinks, including that by athletes. However, the pH values of sports drinks vary from 3 to 4. In the present study, we used a sports drink with a pH of approximately 4.0. Decreases in salivary pH and buffering capacity were found in conditions 4 (intake of sports drink) and 5 (intake of sports drink and food). In contrast, the salivary pH and buffering capacity during and after exercise in condition 2 (intake of mineral water), did not decrease compared with before exercise. From the point of view of preventing an increase in the risk of dental caries, our study results show that mineral water is the best source of fluid intake.

Physical and chemical factors of foods stimulate the oral mucous membranes and tongue surface, inducing salivary secretion in association with meals. Therefore, salivary pH values generally increase immediately after meals [13]. In this study, the salivary flow rates in conditions 3 and 5 were similar. Nanba et al. showed that the salivary secretion-dependent variations in salivary pH values were more influenced by chemical factors than physical factors of food [13]. For example, a study reported that salivary pH values after the meal returned to the original values within 35 min after eating a rice ball [13]. However, after eating a sandwich, the values after the meal returned to the original values within 15 min. In the present study, the salivary pH and buffering capacity after exercise was lower in the case of exercise with intake of sports drink and food, than with intake of mineral water and food. With regard to the risk of dental caries and erosion, consumption of mineral water with food during sports and exercise is desirable in people who participate in exercise and/or competitions.

Nutrients such as glucose, proteins, amino acids, fat, fatty acids, minerals, electrolytes, and vitamins obtained from ingested food are essential for athletes’ growth, development, and maturation [18]. Carbohydrate supplementation is effective in improving performance and deferring fatigue because glucose is the only source of energy for the brain [19]. Human sweat is known to contain minerals such as Na^+^, Cl^−^, and at least 25 other electrolytes and trace elements [20-22]. Consequently, supplements are necessary to maintain the appropriate distribution of electrolytes in the fluid compartment of the body [21,22]. Because K^+^ has a relationship to muscle fatigue, K^+^ supplementation to athletes during prolonged sports and exercise by administering nutritional supplements like bananas is considered necessary [23]. Moreover, they contain many nutrients such as water, protein, carbohydrates, Mg^2+^, and K^+^, the levels of which are three times as high in bananas as in apples [24]. Therefore, in order to maintain a proper amount of electrolytes, athletes should take nutritional supplements during sports and exercise. In case of being unable to taking them during sports and exercise, they should do as early as possible after finishing the activities.

**Figure 2:**
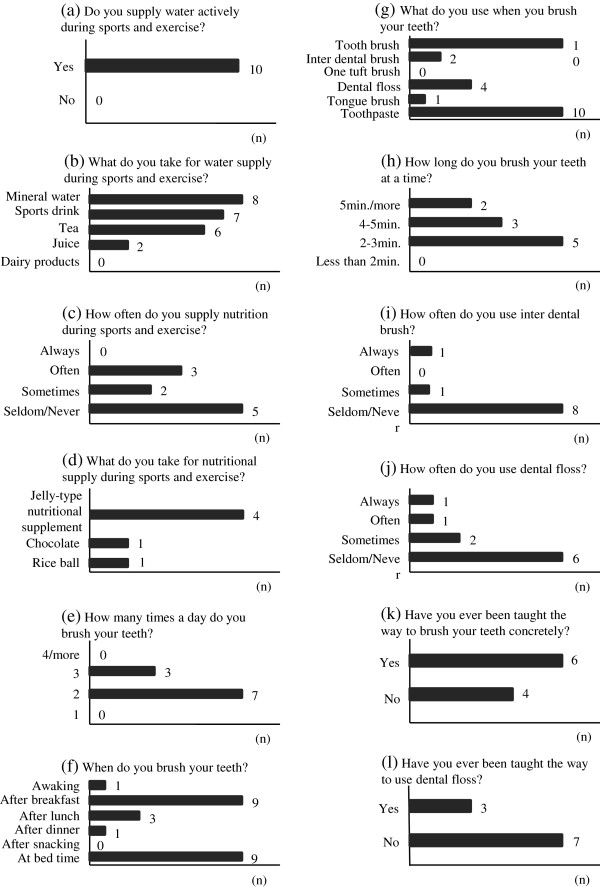
**Questionnaire and frequency related to intake of fluids and food during exercise and sports, and oral health behavior.** Histograms showing the number of responders to each of the questions.

In the present study, 10 participants answered a questionnaire related to the intake of fluids and food during exercise and sports as well as oral health behavior Figure 2. According to the results of the questionnaire, all participants consumed fluids during sports and exercise. Most of them said they drank mineral water or a sports drink. The next most common fluid was tea (green tea and barley tea). Approximately 30% participants who said that they had only tea and/or mineral water during sports and exercise did not consider the fluid intake as food intake but as consumption for quenching their thirst. Half of the participants answered that during exercise, they eat food often or occasionally, and that they liked jelly-type nutritional supplements (for example, Wider In Jerry, from Morinaga & Co., Ltd., Tokyo, Japan). Thus, our study results indicate that 70% participants used sports drinks, jelly-type nutritional supplements, chocolate, and/or rice balls as the preferred method of food intake during sports and exercise.

According to a survey of dental diseases in 2005 in Japan, 50% and 21% participants brushed their teeth two and three times a day, respectively [25]. In the present study, 70% and 30% of the participants brushed their teeth two and three times a day, respectively. The combination of after breakfast and at bedtime accounted for the largest number of them. Therefore, the daily frequency brushing teeth in the present study participants was above the national average. Although 70% participants used sports drinks and half of them nutritional supplements during sports and exercise, none brushed their teeth after snacking. The present study indicated that the risk of dental caries could increase as a result of the conditions of water and nutritional supplementation; therefore, we should pay more attention to oral health care.

There are many adjunctive devices for tooth brushing such as dental floss, interdental brushes, one-tuft brushes, tongue brushes, and toothpaste. Tooth brushing is not sufficient for plaque control, and daily dental flossing has been emphasized for plaque control of proximal surfaces [26]. The American Dental Association reported that up to 80% of plaque might be removed by dental flossing [27]. The present study results revealed that only 10% of the participants used dental floss every day, and indicated that dental flossing is not accepted as a common oral health behavior yet. In addition, the questionnaire survey results indicated that 60% participants had been taught how to brush their teeth, and that only 30% participants had been taught how to use dental floss. Thus, dentists and dental hygienists should help people understood the importance of dental floss for tooth care and the proper way to use dental floss.

## Conclusion

The present study’s results indicated that adequate hydration during sports and exercise decreased salivary secretion and increased the risk of dental caries and erosion. However, during bicycle ergometer exercise, intake of sports drinks and foods were shown to significantly influence the oral circumstances, salivary pH, and buffering capacity, and increased the risk of dental caries and erosion. Therefore, from the point of view of the risks of dental caries and erosion, we advise that people who participate in exercise and competition should consume mineral water along with food during sports and exercise. Individuals consuming a sports drink should pay special attention to their oral health care by measures such as rinsing out their mouth or brushing their teeth after sports and exercise. Dentists and dental hygienists also should inform athletes, laypeople, and coaches that intake of sports drinks and food during sports and exercise might increase the risks of dental caries and erosion.

## Competing interests

The authors declare that they have no competing interests.

## Authors’ contributions

MT participated in the design of the study, carried out the experiment and drafted the manuscript. TT performed the coordination and data analyses of the study, and helped to draft the manuscript. KS participated in the design of the study. YT participated in data arrangement. TU conceived of the study and participated in its design. All authors read and approved the final manuscript.
